# Age-Friendly and LGBTQ2+ Friendly Community Initiatives in Canada: Preliminary Findings

**DOI:** 10.1093/geroni/igab046.1542

**Published:** 2021-12-17

**Authors:** Emma Whitehouse, Kimberley Wilson, Arne Stinchcombe

**Affiliations:** 1 University of Guelph, Guelph, Ontario, Canada; 2 Brock University, St. Catharines, Ontario, Canada

## Abstract

Age-Friendly Communities (AFC) initiatives are gaining momentum in Canada and around the globe with many communities making commitments to becoming age-friendly. Aging lesbian, gay, bisexual, transgender, queer, and two-spirit (LGBTQ2+) Canadians are a diverse subpopulation whose social histories and contexts may not have been considered in such initiatives. In response, many community-level organizations have created programs and supports for older LGBTQ2+ persons. Through a survey and environmental scan, we sought to identify and profile such initiatives. In addition, in-depth interviews were held with representatives from community groups to ascertain how community leaders construct and define communities that are both age and LGBTQ2+-inclusive. Participants were also asked to reflect on how their sense of community and support was impacted by the Covid-19 pandemic. The findings indicated that many supports for LGBTQ2+ older adults emerged outside of formal AFC initiatives and in response to a perceived normativity among services for older adults.

